# Ligament reconstruction combined with osteotomy: Case report

**DOI:** 10.1097/MD.0000000000043419

**Published:** 2025-08-01

**Authors:** Yaomin Wang, Heng Cao, Xinlong Chen, Qiang Ren, Jian Li, Hui Shi

**Affiliations:** a Department of Bone Joint, Binzhou Medical University Hospital, Binzhou, China.

**Keywords:** anterior cruciate ligament, distal femoral osteotomy, graft fixation, medial patellar retinaculum, reconstruction

## Abstract

**Rationale:**

Valgus deformity of the knee joint is caused by congenital anatomical abnormalities. The occurrence of an anterior cruciate ligament (ACL) tear in conjunction with a medial patellofemoral ligament (MPFL), alongside knee valgus deformity, is exceedingly rare in clinical practice.

**Patient concerns:**

A 30-year-old female presented with congenital valgus knee. The left knee joint was immobile due to pain and swelling after trauma. The patient had a history of hypertension.

**Diagnoses:**

The patient underwent an MRI of the left knee joint at a local hospital, and was diagnosed with ACL injury of the left knee, medial patellofemoral ligament (MPFL) injury of the patella, and congenital valva deformity.

**Interventions:**

The patient underwent simultaneous reconstruction of the ACL and MPFL, as well as a distal femoral osteotomy.

**Outcomes:**

The patient experienced significant postoperative improvement, with marked pain relief. The knee remained stable, and functional recovery was observed.

**Lessons:**

Failure to adequately address the stability of the patella following the rupture of the MPFL may result in increased stress on the ACL graft, thereby heightening the risk of graft failure. Additionally, valgus deformity can impose abnormal mechanical loads on the ACL graft, substantially elevating the likelihood of complications such as articular cartilage degeneration and meniscal injury. Usually, we need accurate guidance on imaging and evaluation of surgical options to successfully complete the surgery.

## 1. Introduction

The anterior cruciate ligament (ACL) is among the most frequently injured ligaments in the human body. It serves as a primary constraint against tibial plateau advancement, knee hyperextension, and tibial rotation. ACL injuries typically do not occur in isolation; rather, they are often associated with meniscal injuries in approximately 50% of cases and collateral ligament injuries in about 20% of cases.^[1]^ ACL reconstruction is the most commonly performed knee surgery and is typically associated with a high success rate. The success of this surgical procedure is primarily influenced by several factors, including the acquisition of the tendon, the precise location of the tunnels, the tension applied to the graft during fixation, and the types of fixation used for both the femur and tibia.^[2–4]^

The medial patellofemoral ligament serves as the primary limiting factor for lateral translation of the patella during knee flexion ranging from 0 to 30 degrees,^[5]^ The occurrence of instability and dysfunction in the knee joint following an injury may necessitate treatment options such as reconstruction or repair. Valgus deformity is classified into 2 categories: physiologic and pathological. Prolonged valgus deformity can result in heightened instability of the ACL.

This report presents a case of ligament reconstruction and orthopedic osteotomy of a traumatic knee joint, highlights the technical and administrative challenges, and demonstrates successful outcomes following surgical treatment.

## 2. Case presentation

A 30-year-old female presented with left knee joint pain, swelling, and limited mobility due to trauma 1 day ago. After completing the auxiliary examination in the local hospital, it is recommended to be referred to our hospital for further management and treatment. The patient had a history of hypertension and was not systematically treated.

Upon admission, the patient reported persistent joint pain in the left knee and difficulty moving. No fever or chills. Physical examination revealed a positive patellar apprehension test, a positive Lachman test, and a positive McMurray–Fouche test, while the range of motion in the left knee joint was found to be normal. Beighton score was within the normal range. After thorough communication and inquiry with the patient and his family, surgical intervention was decided.

With the acquisition of signed informed consent and the administration of combined epidural anesthesia, the patient underwent arthroscopically assisted reconstruction of the ACL and the medial patellofemoral ligament (MPFL), in addition to a distal femoral osteotomy and orthopedic internal fixation.

A double-sided osteotomy was executed while preserving the lateral cortex. Preoperative radiographic assessment confirmed significant valgus malalignment:

- Hip–knee–ankle angle (HKA): 175.42° (normal range: 177°–183°)
- Mechanical axis deviation (MAD): 16.14 mm (lateral deviation, normal range: ±10 mm)

**Figure 1.**
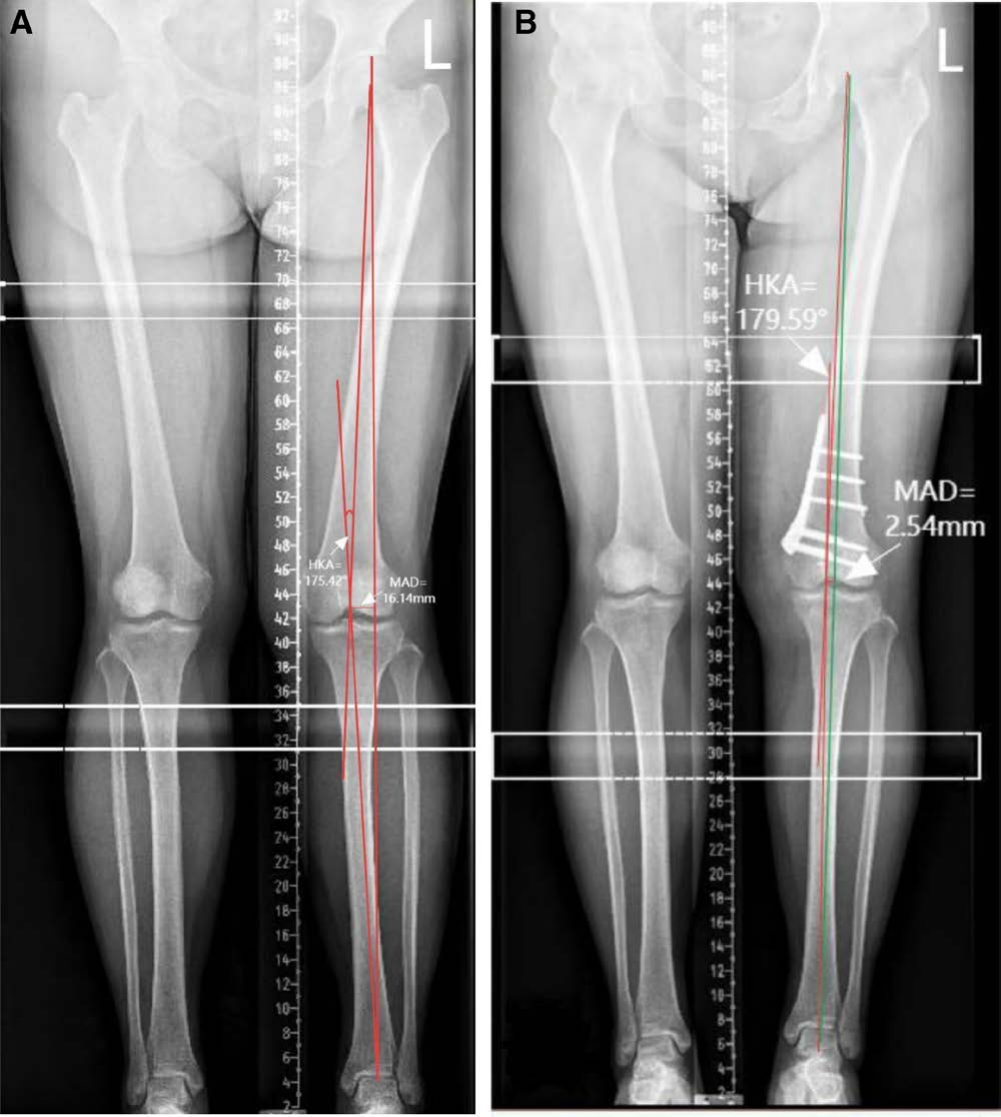
(A–D) Preoperative axial (A and B) and lateral (C and D) radiographs of the left knee demonstrating patellofemoral malalignment and significant lateral patellar displacement.

The patient underwent a distal femoral osteotomy. Osteotomy biomechanical calculation was based on Hernigou 2010 trigonometric correction chart (Table 2), The specific parameter selection process is as follows:

- Preoperative HKA: 175.42°
- Target HKA: 180°
- ΔHKA = (Preoperative HKA)-(Target HKA) = 4.58° (4°and 5°)
- Mediolateral Femoral Diameter: 65 mm (measured via CT, Fig. 1A and B)
- Interpolating between depth values:
- 4° depth = 5 mm
- 5° depth = 6 mm
- Calculated depth = 5 + (4.58–4)×/ = 5.58 mm → rounded to 5.6 mm

This methodology ensured precise wedge resection matching the required 4.58° HKA correction. Angle selection: 5.6° (from Hernigou chart for 65mm diameter and ΔHKA = 4.58°).

### 2.1. A biplanar closed-wedge osteotomy was performed with 5.6 mm depth and 5.6° correction angle

**Figure 2.**
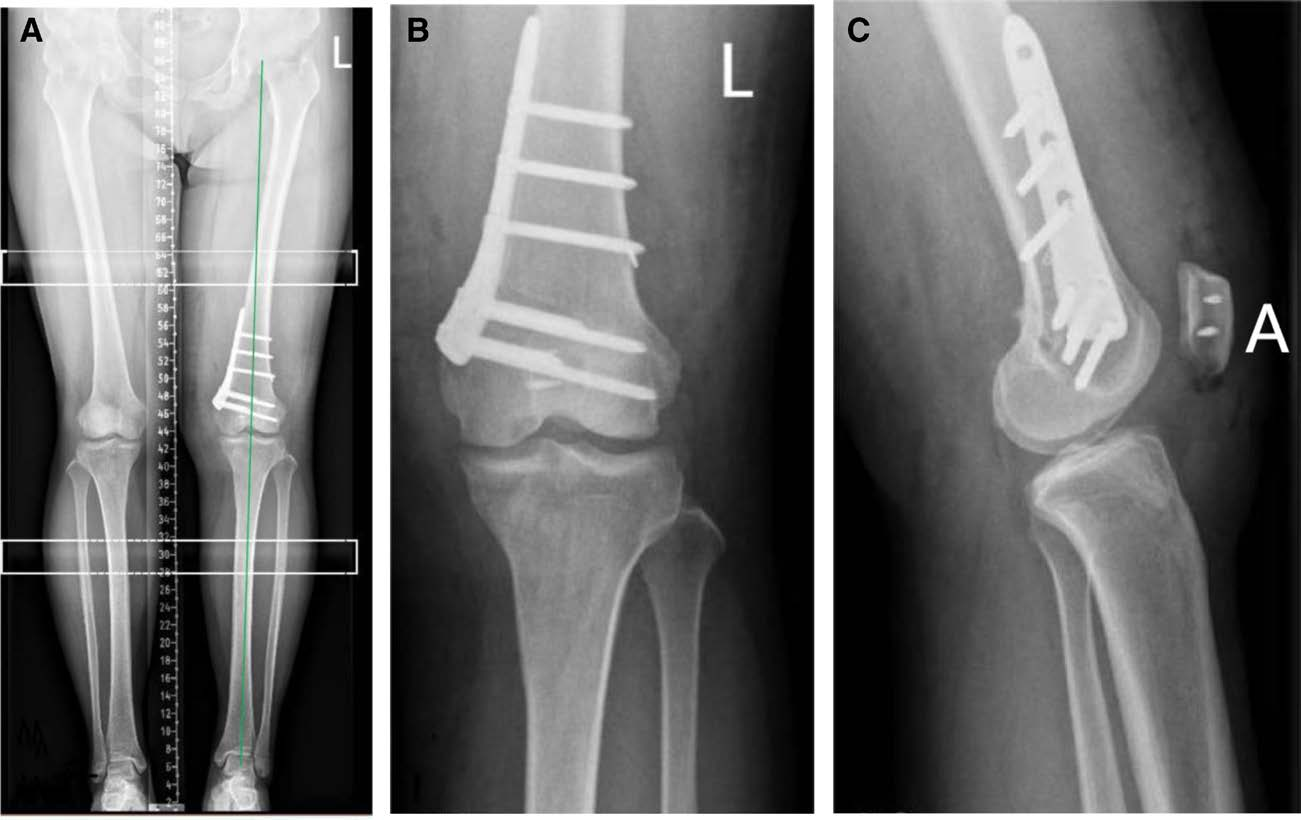
(A and B) Preoperative (A) and postoperative (B) schematic representations of the biplanar distal femoral closed-wedge osteotomy. Planned wedge depth: 5.6 mm; correction angle: 5.6°.

**Figure 3.**
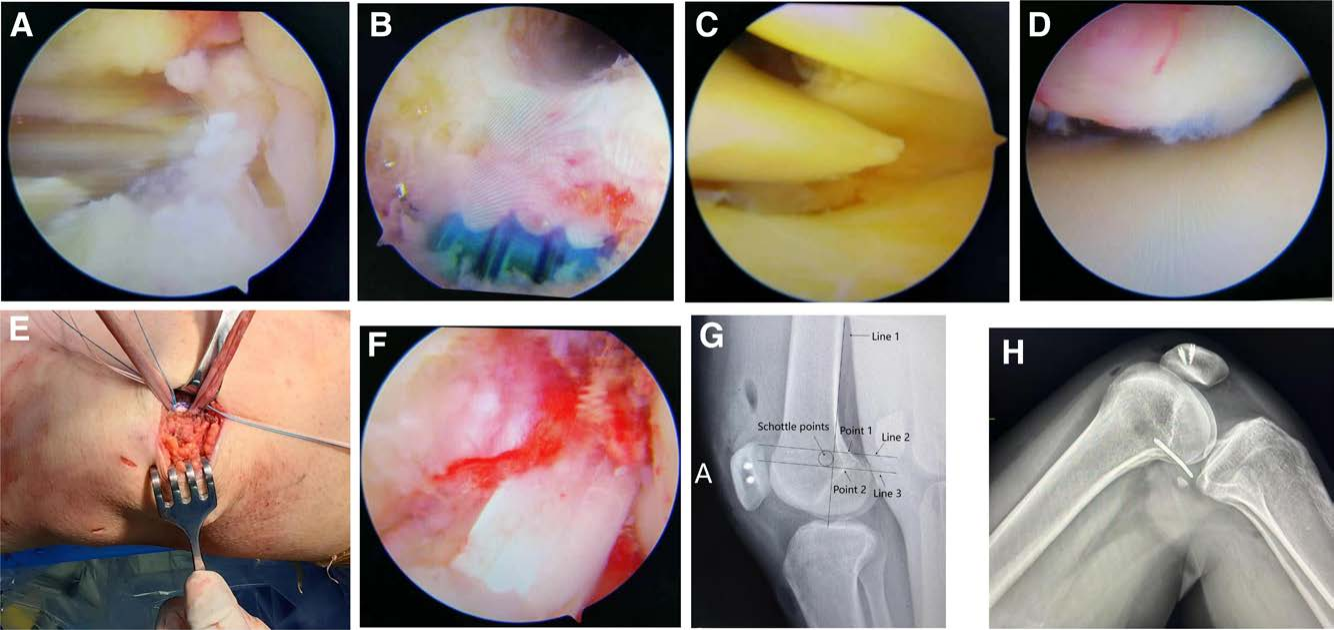
(A–C): Intraoperative fluoroscopic images demonstrating correction of lower limb alignment following distal femoral closed-wedge osteotomy with locking plate fixation. Anteroposterior (A), lateral (B), and full-length weight-bearing (C) views.

X-ray fluoroscopy confirmed that the lower limb force line was appropriately corrected and that the internal fixation was secure (Figs. 2A, B, and 3A–C).

During the arthroscopic examination, it was determined that the ACL was completely torn, and the body of the lateral meniscus was transected near the hiatus of the popliteal tendon. Notably, the articular cartilage exhibited no significant damage. The thin femoral tendon and a portion of the long peroneal tendon were dissected, and the femoral tunnel was created. The tibial tunnel locator was adjusted, and a traction line was introduced from the tibial tunnel through the femoral tunnel. During the operation, a cylindrical Graft measuring device (such as Arthrex® Graft Sizer) was used to confirm that the diameter of the ACL graft was 8mm. A total internal suture of the ruptured meniscus was performed, resulting in a firm and stable suture. No intraoperative complications or adverse events were observed. Under microscopic examination, the stroke tension of the graft, assessed with a probe hook, was found to be satisfactory, and there was no impingement in the intercondylar fossa. The diameter of the ACL graft during the operation was 8mm, and no InternalBrace or other enhancement techniques were used. The tibial insertion point of the ACL graft is located at the center of the footprint area.

**Figure 4.**
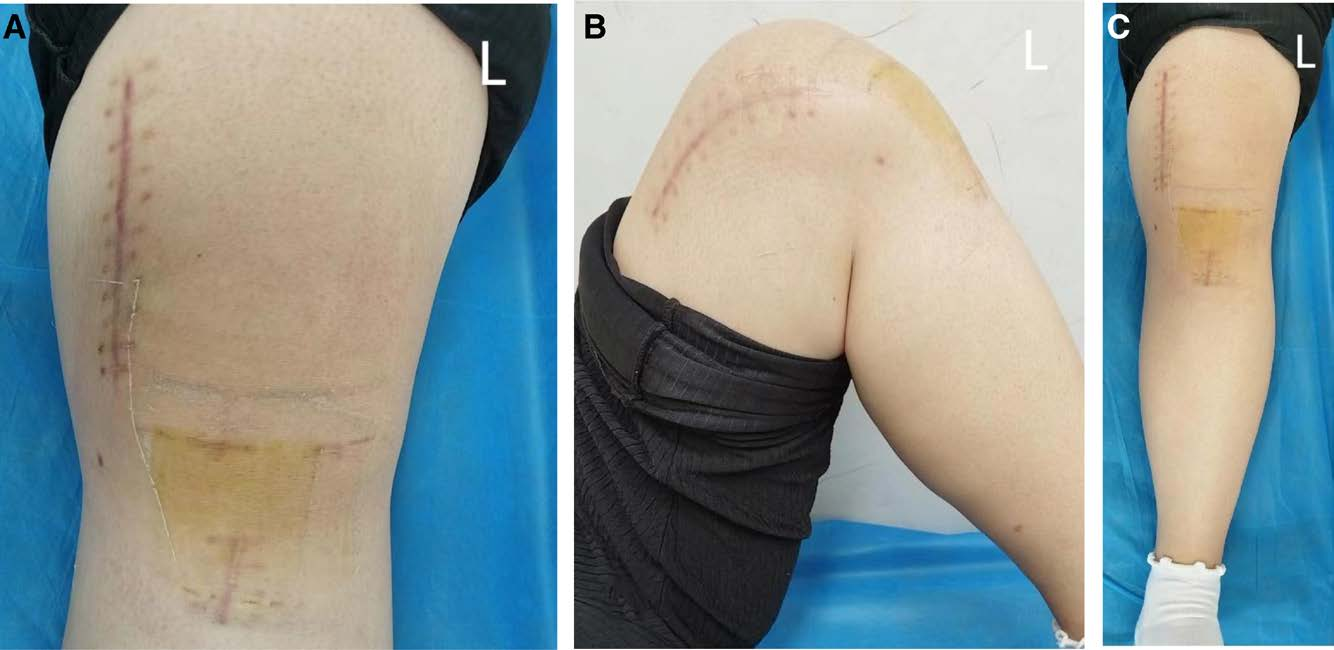
(A–H): Arthroscopic views during simultaneous ACL and MPFL reconstruction. (A and B) ACL femoral tunnel preparation and graft passage. (C and D) MPFL femoral tunnel preparation and graft positioning. (E) Intraoperative ACL tibial insertion point. (F) Final fixation and tensioning of ACL and MPFL grafts. (G) The zhidianShottle method was used to determine the femoral insertion point. (H) The femoral points were determined by intraoperative image navigation. ACL = anterior cruciate ligament, MPFL = medial patellofemoral ligament.

The medial patellofemoral ligament (MPFL) was subsequently reconstructed, the reconstruction points for the patellar tunnel were established at the middle and upper one-third and one-half of the internal patellar margin, respectively. Two 3.6 mm metal anchors were inserted into the patellar tunnel, and the semitendinosus tendon was introduced into the patellar bone tunnel. The graft was subsequently introduced into the femoral tunnel, and tension was applied to the graft within the knee joint to identify the isometric point during flexion and extension activities. Postoperatively, the knee exhibited satisfactory flexion and extension capabilities, appropriate tension, and the drawer test yielded negative results prior to the postoperative assessment. Lateral X-ray films were used to locate the femoral tunnel through 3 reference lines (Fig. 4A–F).

The positioning points (Schottle points) are located 1.3 mm in front of the extension line of the posterior femoral cortex (Line 1), 2.5 mm at the distal end of the line (Line 2) passing through the junction of the posterior condyle of the femur and the posterior cortex and perpendicular to the extension line of the posterior femoral cortex, and 5.5 mm at the proximal end of the line (Line 3) passing through the posterior Angle of the Blumensaat line and perpendicular to the extension line of the posterior femoral cortex. Among them, line 1 determines the front-back position of the Schottle point, while lines 2 and 3 determine the near and far position of the Schottle point (Fig. 4G). During the operation, the equal-length points of the femur of the MPFL graft were confirmed through image navigation. To confirm the isometric points of the femur in MPFL and measure the changes in the length of the graft, fluoroscopy-guided real-time distance monitoring technology was adopted during the operation. The specific method is as follows: two miniature impenetrable X-ray marker needles (metal needles) were accurately implanted at the planned positions on the femoral side (i.e., the planned Schottle point area) and the patellar side (near the patellar tunnel fixation point), as shown in Figure 4H. Subsequently, under the intraoperative C-arm machine fluoroscopy guidance, the knee joint was continuously and passively flexes from 0° to 90°. During this flexion and extension activity arc (0°–90°), the Euclidean spatial distance (representing the simulated graft length) between the tip of the 2 marking needles was measured and recorded in real time and dynamically using the distance measurement software attached to the fluoroscopy system. Repeat the measurement 3 times and take the average value to ensure reliability. By continuously observing the dynamic changes of this distance throughout the entire flexion process, the femoral implantation point with the smallest change in graft length (<2 mm) was finally selected as the optimal isometric point (Fig. 4H shows the positional relationship between the final positioning point on the femoral side and the marker needle on the patellar side, which is a confirmation figure for the measurement results).

On the first postoperative day, patients are advised to initiate functional exercises, including quadriceps isometric exercises and partial weight-bearing activities utilizing a walker.

#### 2.1.1. Schedule of weight-bearing progress

Day 1 after the operation: with the assistance of a walker, toe touch weight bearing was initiated. 0 to 6 weeks after the operation: partial weight bearing, with a target of 25% to 33% of body weight, using a walker or crutches. The degree of weight-bearing is gradually increased according to the patient’s tolerance, pain and swelling conditions, and strictly follows the imaging assessment of osteotomy healing (usually rechecked with X-rays at 6 weeks). 6 to 12 weeks after the operation: according to the osteotomy healing shown by the X-ray, gradually transition to full-weight bearing. Typically, 50% weight is achieved at 6 to 8 weeks, 75% at 8 to 10 weeks, and full weight at 10 to 12 weeks. Continue to use the walker or crutches until the gait is normal and there is no pain or limping. Twelve weeks after the operation: full-weight-bearing and walking without crutches.

Imaging assessment should be comprehensively judged based on the following clear criteria. Only when all are met can the weight be gradually increased to full weight:

The quality and quantity of callus formation: continuous and bridging callus formation should be observed at the osteotomy end, with a progressive increase in callus density.

Stability of internal fixators: confirm that all steel plates, screws and bone graft areas (if used) remain stable. There were no signs of any imaging loosening.

Maintenance of osteotomy position: measured on X-ray films in the standing position/weight-bearing position, the target correction Angle should not be significantly lost compared with the early postoperative period. Ensure that the corrective effect is maintained.

The boundary of the osteotomy area: the osteotomy line should start to become blurred rather than sharp and clear, indicating that the bone healing process is underway.

If the X-ray assessment at 6 weeks fails to fully meet the above standards (such as poor callus formation, signs of loose internal fixation, loss of force lines, etc), the partial weight-bearing state should continue, and the X-ray films should be rechecked every 2 to 4 weeks until the standards are met before gradually transitioning to full-weight-bearing.

#### 2.1.2. Limitation of joint range of motion

Zero to two weeks after the operation: use a range of motion brace for the knee joint and lock it in a fully extended position (0°). It is allowed to perform 0° isometric quadriceps contractions and straight leg raises under the protection of braces. Two to 4 weeks after the operation: the brace is unlocked, allowing passive/assisted active range of motion training (0°–60°). 4 to 6 weeks after the operation: the range of motion increases to 0° to 90°. 6 to 8 weeks after the operation: the range of motion increases to 0° to 120°. Eight to twelve weeks after the operation: strive to achieve full range of motion (0°–130°+).

#### 2.1.3. Special protective measures for MPFL reconstruction

Zero to six weeks after the operation: use hinged knee braces with Patellar stabilization pads or anti-lateral displacement designs to strictly limit the lateral movement of the patella. All rehabilitation training must be carried out under the protection of braces. Avoid performing any movements or training that may increase the lateral stress of the patella in the early postoperative period (at least the first 6 weeks), such as: lateral kicking or sliding training is prohibited. Avoid keeping the knee joint in a slightly flexated position (20°–40°) for a long time, as this is the most unstable Angle range of the patella.

Passive range of motion training and early knee flexion exercises (such as bedside leg hanging) should be strictly controlled by the therapist to ensure that the patellar trajectory is centered and to avoid violent external pushing of the patella. Six to twelve weeks after the operation: under the protection of braces, controllable and low-intensity Patellar mobilizations can be initiated, with a focus on medial sliding (which should be carried out under the guidance of a therapist). Continue to avoid severe lateral stress. Twelve weeks after the operation: based on the clinical assessment (such as patellar stability tests) and functional recovery, more challenging closed-chain movements (such as micro-squats) will be gradually introduced, but the patellar trajectory still needs to be monitored. It usually takes 6 to 9 months or even longer to fully resume sports such as running, jumping, and sharp turns and stops.

**Figure 5.**
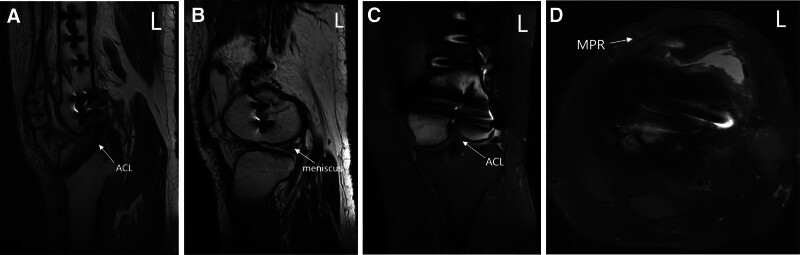
(A–C): Clinical photographs at 4-mo follow-up demonstrating active knee range of motion: full extension (A), mid-flexion (B), and maximal flexion (C).

**Figure 6.**
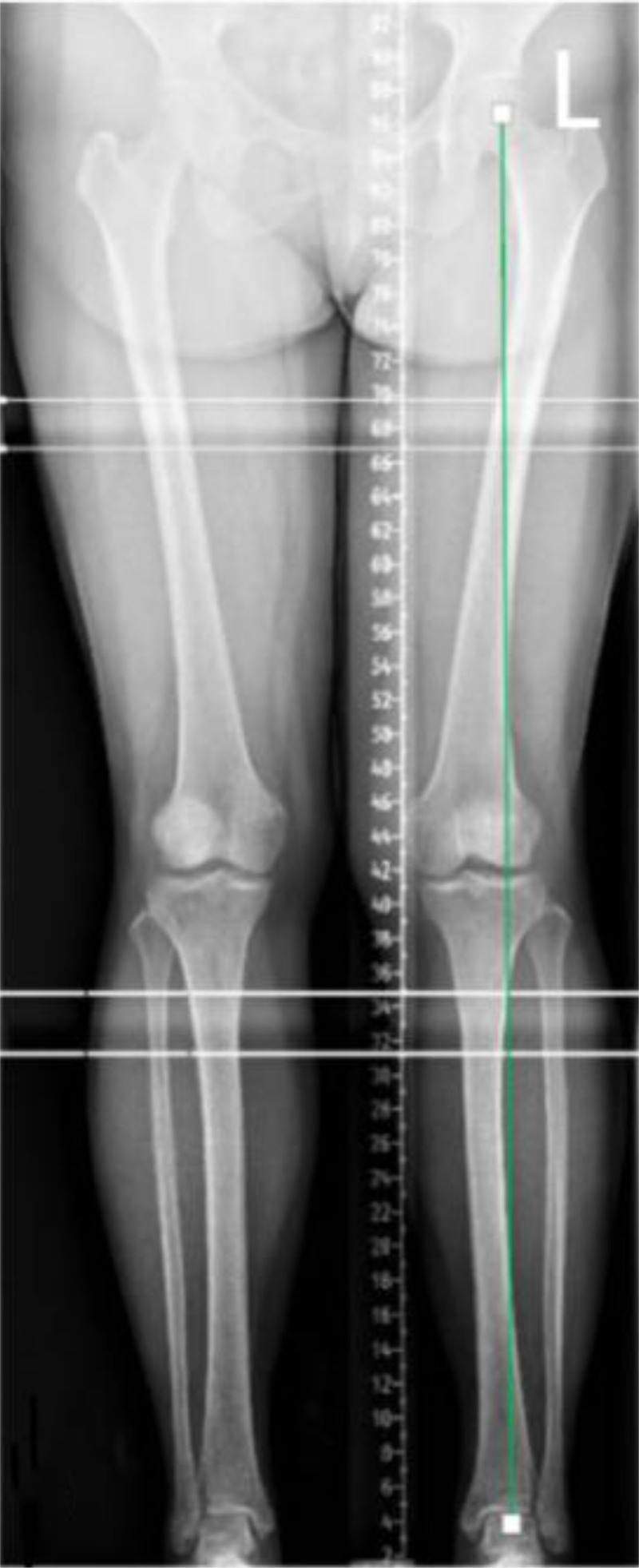
(A–D) Postoperative (4-mo) MRI of the left knee. (A and B) Sagittal and coronal views showing intact ACL graft with appropriate tension. (C and D) Axial and coronal views showing intact MPFL graft with appropriate tension. MPFL = medial patellofemoral ligament, MRI = magnetic resonance imaging.

During 1 year of follow-up, the patient’s knee movements had largely returned to normal (Fig. 5A–C). The results of the anterior drawer test were negative, as were those of the Lachman test. Magnetic resonance imaging (MRI) of the knee indicated that the reconstructed ACL and the medial patellofemoral ligament (MPFL) exhibited appropriate morphological tension, with no significant adverse reactions observed (refer to Fig. 6A–D). The intervention measures and assessment results of the patients at different time periods after the operation were recorded respectively (Table 1).

**Table 1:** Postoperative follow-up schedule.

Time (after the operation)	Intervention measures	Evaluation result
Day 1	- Start isometric contraction training of the quadriceps - Partial weight-bearing activities with the assistance of a walking aid	- Vital sign monitoring - Pain assessment (VAS score) - Wound examination (no bleeding or infection)
Day 7	- Wound dressing change - Guide active ankle pump training	- Assessment of the degree of joint swelling - Active flexion range of the knee joint (Target: 0°–30°) - Pain control effect (medication adjustment)
The first month	- Physical therapy intensification (progressive resistance training) - Full-weight walking training (depending on tolerance)	- Range of motion of the knee joint (target: 0°–90°) - Preliminary assessment of the front drawer test/Lachman test - X-ray examination (for evaluating the healing of the osteotomy end and the position of internal fixation)
The third month	- Dynamic balance training (such as standing on 1 leg - Low-intensity aerobic exercise (such as stationary cycling)	- Knee joint stability test (negative axial displacement test) - Muscle strength assessment (quadriceps/hamstrings ratio)
The sixth month	- Functional exercise training (such as going up and down stairs, jogging) - Proprioception training	- Range of motion of the knee joint (Target: above 0°–120°) - The IKDC score/Lysholm score was used to assess functional recovery - CT (Evaluation of osteotomy correction Angle and force line)
The first year	- Fully resume daily activities and low-risk sports (such as swimming) Long-term follow-up plan formulation	- Comprehensive stability test of the knee joint (negative front drawer/Lachman test) - MRI (evaluation of ligament morphology and tension, see Fig. 5A–D) - Patient subjective satisfaction survey

CT = computed tomography, IKDC = international knee documentation committee subjective knee, MRI = magnetic resonance imaging, VAS = visual analogue scale.

Furthermore, in this case report, the long-term effect data of harvesting the peroneus longus tendon on ankle stability were analyzed using the postoperative AOFAS functional scale (Table 2). The data of this scale clearly indicate that using the autologous peroneus longus tendon to reconstruct the ankle ligament can effectively restore and maintain good ankle stability and function for a long time. The postoperative functional score significantly improved over time and reached a high level close to normal or completely normal at 6 months and 1 year after the operation.

**Table 2:** AOFAS ankle-hindfoot scale.

Time (after the operation)	Score	Explanation
Day 1	20	Severe pain or functional impairment
Day 7	40	Severe pain or functional impairment
The first month	67	Moderate pain or functional limitation
The third month	87	Mild pain or basically normal function
The sixth month	95	Painless and functionally normal
The first year	100	Painless and functionally normal

AOFAS = ankle-hindfoot scale

Preoperative imaging of the patient has been perfected.

**Figure 7.**
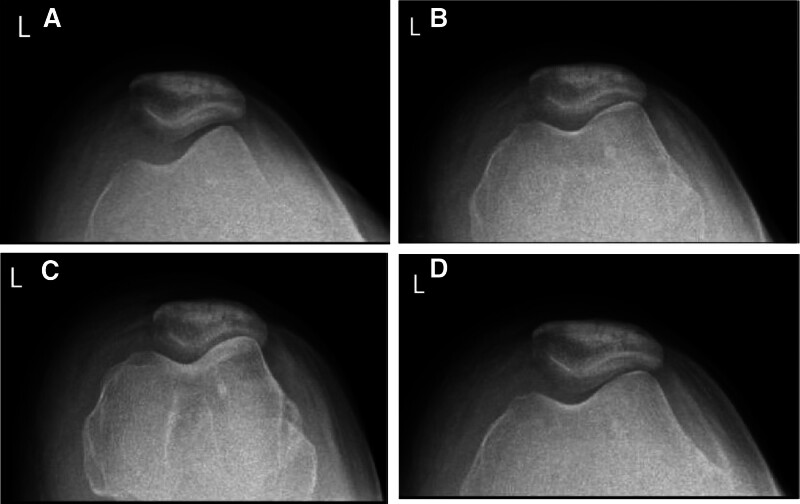
Preoperative standing full-length anteroposterior radiograph of the lower limbs demonstrating significant valgus malalignment (HKA: 175.42°, MAD: 16.14 mm lateral deviation). MAD = mechanical axis deviation, HKA = Hip-knee-ankle angle.

Plain radiographs indicated that the force line of the patient’s left lower limb was positioned outside the midpoint of the tibial plateau, which is a characteristic manifestation of genu valgus (Fig. 7).

Plain radiographs indicate inadequate patellofemoral alignment and considerable lateral displacement of the patella (Fig. 1A–D).

Computed tomography (CT) for tibial tubercle-trochlear groove (TT-TG) distance measurement:

#### 2.1.4. Landmark identification

1. Trochlear groove (point G): on the axial slice demonstrating the deepest point of the trochlear sulcus, the deepest point in the cartilaginous or bony trochlear groove was identified.
2. Tibial tubercle (point T): on the axial slice demonstrating the most prominent aspect of the tibial tubercle, its peak was identified.

Reference Axis: a single, common reference line was established using the posterior femoral condylar line (PFCL). The PFCL was drawn connecting the most posterior points of the medial and lateral femoral condyles on the axial slice where both condyles were best visualized.

#### 2.1.5. Measurement technique

1. Two lines perpendicular to the PFCL were drawn: 1 passing through Point G and the other passing through Point T.
2. The distance (in millimeters) between these 2 perpendicular lines was measured. This distance represents the TT-TG distance.
3. Measurements were performed using the calibrated electronic distance measurement tool within the picture archiving and communication system.

**Figure 8.**
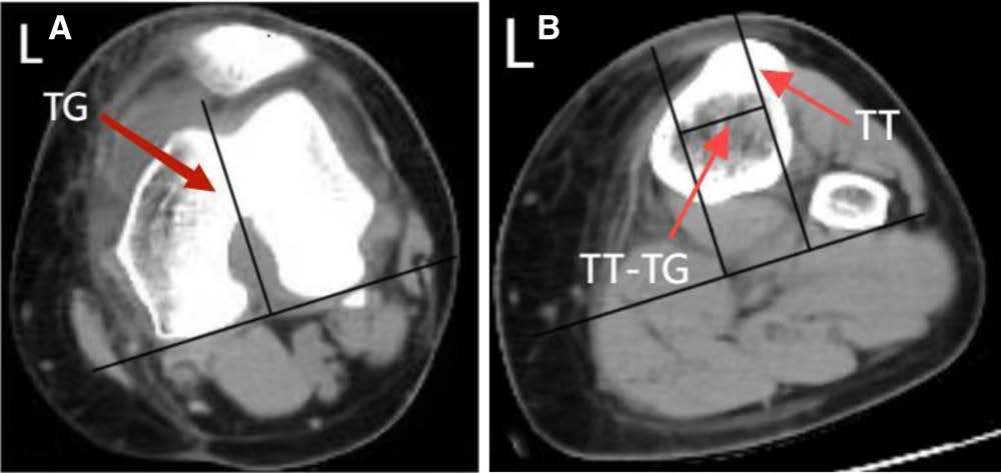
(A and B) Computed tomography measurement of the tibial tubercle-trochlear groove distance (TT-TG distance) according to ISAKOS standards. (A) Axial slice at the level of the deepest point of the femoral trochlear groove (point G). The PFCL is drawn. A perpendicular line to the PFCL passes through Point G. (B) Axial slice at the level of the peak of the tibial tubercle (Point T). The PFCL from (A) is maintained as the common reference axis. A perpendicular line to the PFCL passes through Point T. The TT-TG distance (31 mm) is measured as the distance between the 2 perpendicular lines on superimposed images aligned using the PFCL. Measurement performed using the PACS system electronic distance tool. PACS = picture archiving and communication system, PFCL = posterior femoral condylar line.

Result: a TT-TG distance of 31 mm was recorded, significantly exceeding the ISAKOS-defined pathological threshold of > 20 mm and indicating severe patellar instability (Fig. 8A and B).

#### 2.1.6. Lower limb alignment assessment

Image acquisition: standing, weight-bearing anteroposterior (AP) radiographs of the entire lower limb (hip-knee-ankle, HKA view) were acquired preoperatively according to standardized protocols to assess the mechanical axis.

#### 2.1.7. Measurements

1. Hip–knee–ankle angle (HKA): the angle formed by the mechanical axis of the femur (line connecting the center of the femoral head to the center of the knee) and the mechanical axis of the tibia (line connecting the center of the knee to the center of the talar dome). Normal range: 177° to 183° (physiological varus 0°–3°). Valgus is indicated by an angle > 183°.
2. MAD: the perpendicular distance (in millimeters) from the mechanical axis line of the lower limb (line connecting the center of the femoral head to the center of the talar dome) to the center of the tibial spine tips at the knee joint level. Normal range: ±10 mm (medial deviation indicates varus, lateral deviation indicates valgus).

Result: preoperative radiographic assessment confirmed significant valgus malalignment:

- Hip–knee–ankle angle (HKA): 175.42° (indicating valgus deformity)
- MAD: 16.14 mm (lateral deviation)

**Figure 9.**
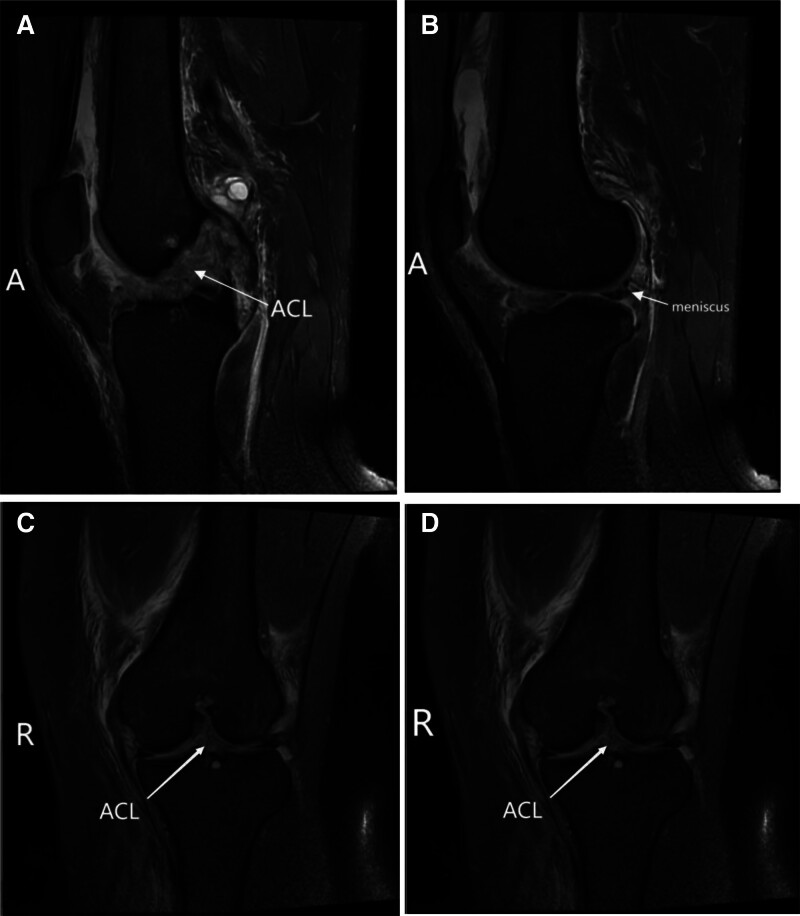
(A–D) Preoperative MRI of the left knee. (A and B) Sagittal and coronal views demonstrating complete tear of the ACL at its femoral origin (arrow). (C and D) Axial views demonstrating tear of the MPFL near its patellar insertion (arrow) and lateral patellar displacement. ACL = anterior cruciate ligament, MPFL = medial patellofemoral ligament, MRI = magnetic resonance imaging.

The MRI of the knee demonstrated a partial tear of the ACL at its femoral attachment site, as well as an injury to the medial patellofemoral ligament (MPFL) of the left knee and damage to the posterior horn of the lateral meniscus (Fig. 9A–D).

## 3. Discussion

The occurrence of an ACL injury in conjunction with a medial patellofemoral ligament (MPFL) injury, alongside knee valgus deformity, is exceedingly rare in clinical practice. There is a limited number of articles that have addressed the treatment modalities for this condition, and even fewer studies have focused on the reconstruction of the ACL and the medial patellofemoral ligament (MPFL), as well as the correction of valgus deformity. The internal patellar support band serves as a crucial structure for stabilizing the patella and preventing dislocation, with its functionality being particularly significant when the knee is flexed between 0° and 30°.^[6]^ The medial patellar ligament system is composed of 3 layers superimposed.^[7]^ The first layer is the superficial ligament; The second layer is the MPFL and the medial collateral ligament; The third layer corresponds to the medial patellotibial ligament (MPTL) and the medial patellosemilateral ligament (MPML). From a biomechanical perspective, MPFL is the deep and independent ligament structure of MPR, and its starting and ending points (from the medial epicondyle of the femur to the upper 1/3 of the medial side of the patella) are quantified, in contrast to the extensive fibers of superficial MPR, and the role of MPFL in patellar stability accounts for 50% to 60%.^[8–10]^

**Figure 10.**
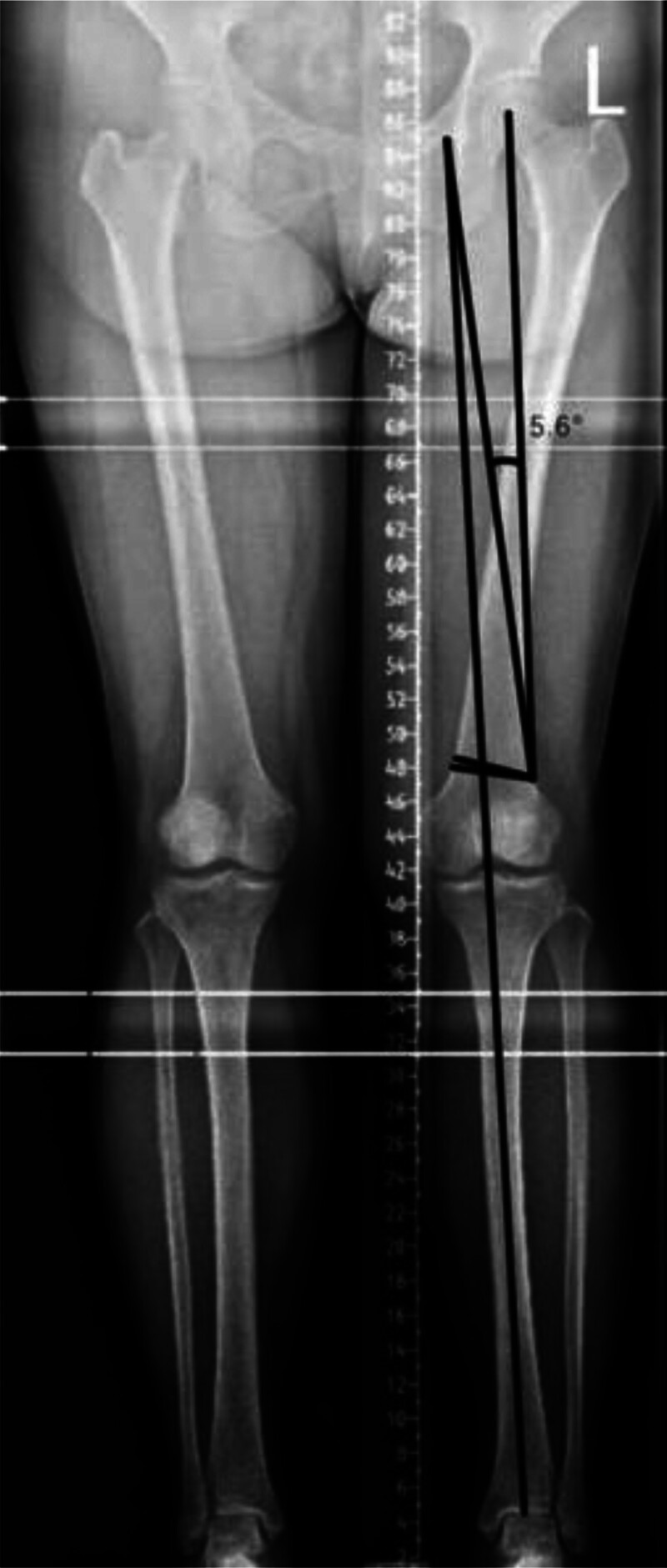
(A and B) Patellar subluxation was performed before the operation (A), and the corresponding relationship between the patellofemoral joint was good after the operation (B).

The medial patellofemoral ligament (MPFL) is a supporting band tissue that connects the medial epicondyle of the femur and the medial edge of the patella. Although the MPFL is very thin, its average tensile strength is 208 N. The contribution of the MPFL to resisting lateral patellar suprabdislocation is the greatest in the extended knee joint.^[5]^ This case reports that the patella of this patient was in a sublingual state before the operation, and the correspondence between the patellofemoral joint was poor. Through MPFL reconstruction, the correspondence between the patellofemoral joint was improved (Fig. 10A and B).

Knee valgus deformity is a significant risk factor for ligament injuries. This paper presents a case study of a patient with knee valgus deformity who experienced ruptures of the ACL and the medial patellofemoral ligament (MPFL) following trauma. The patient underwent ligament reconstruction, osteotomy, and orthopedic surgery, and demonstrated a favorable recovery postoperatively. It explains why MPFL tears are more likely to lead to recurrent dislocation.

A range of graft options is available for ACL reconstruction, including autografts, allografts, and synthetic materials. Research indicates that while autologous hamstring tendons offer considerable strength, grafts with smaller diameters, particularly those measuring <8 mm, may compromise tensile strength from a biomechanical perspective.^[11]^ Studies have confirmed that within an average follow-up period of 14 months, 1.7% of grafts with a diameter > 8mm required revision, 6.5% of grafts with a diameter ranging from 7.5 to 8mm required revision, and 13.6% of grafts with a diameter of 7mm or smaller required revision.^[11]^ Mariscalco et al^[12]^ also recently showed the important relation between graft size and risk of revision. In grafts that were >8 mm in diameter, the revision rate, which was used as an indicator of graft failure, was 0% for all age groups. However, for patients aged 18 years or younger, the failure rate of a graft 8 mm or less in diameter was 18.3%, whereas patients older than 18 years with grafts 8 mm or less in diameter had a failure rate of 7%. Based on existing research data, the diameter of the graft is also a key factor influencing the repair rate and clinical prognosis after ACL reconstruction. Prioritizing the use of grafts with a diameter >8 mm (such as the combination of the long peroneal tendon and the semitendinosus tendon) can significantly reduce the risk of postoperative re-tear, especially for young patients with incomplete skeletal development or high activity demands. If the diameter of the autologous tendon is insufficient, allograft tendons or artificial ligaments can be considered as supplements, or composite transplantation techniques (such as double-bundle reconstruction) can be used to meet the biomechanical requirements. Future research will still need to evaluate the remedial effects and complication risks of different graft materials (autologous, allogeneic, synthetic) when the diameter is insufficient.

In recent years, autologous peroneal long tendons have gained prominence as grafts for ligament reconstruction following ligament injuries,^[13]^ primarily due to their favorable accessibility. The hamstring tendon, in conjunction with the peroneus longus tendon, may be utilized as a graft for ACL reconstruction.

Macura and Veselko utilized the quadriceps tendon for the reconstruction of the ACL and the medial patellofemoral ligament (MPFL). The deep layer of the tendon was employed for the internal patellar support band, whereas the superficial layer was designated for ACL reconstruction.^[14]^ Steensen et al^[15]^ utilized quadriceps tendon grafts to enhance the reconstruction of the medial patellar retinaculum. Hiemstra et al^[16]^ reported on a cohort of 13 patients, comprising 8 individuals who received allograft and 7 who underwent hamstring autograft procedures for ligament reconstruction. The reconstruction of the ACL was performed initially, followed by the reconstruction of the medial patellofemoral ligament. The ACL was reconstructed utilizing the gracilis tendon in conjunction with a segment of the peroneus longus tendon, while the medial patellofemoral ligament was reconstructed using a graft from the semitendinosus tendon.

The biplanar femoral distal medial wedge closure osteotomy is an appropriate surgical intervention for unicondylar knee joint disease and femoral valgus deformity. Direct bone grafting eliminates the need for bone transplantation and facilitates immediate postoperative weight-bearing and movement of the knee joint. Consequently, this technique is associated with a reduced incidence of complications, aesthetically pleasing incisions, and a favorable prognosis.^[17]^

**Figure 11.**
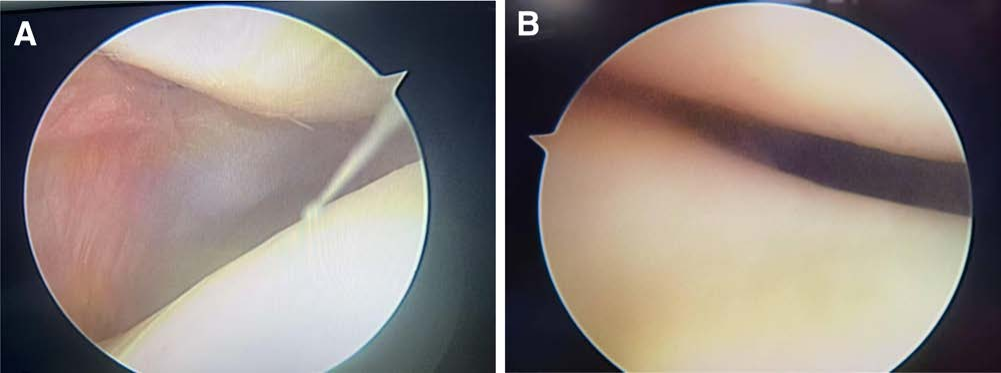
Schematic diagram illustrating the biplanar distal femoral closed-wedge osteotomy technique. Transverse osteotomy (~3/4 femur width). Coronal osteotomy oriented at 100° to the transverse cut. Wedge dimensions: depth 5.6 mm, angle 5.6°.

In this study, a double-plane closed-wedge osteotomy of the distal femur was adopted. The biomechanical basis was based on the osteotomy parameter table and calculation formula proposed by Hernigou et al (2010). This surgical method, by precisely calculating the depth and Angle of osteotomy, can simultaneously correct coronal valgus deformity and sagittal articular line tilt, and avoid secondary joint degeneration after surgery. The quantitative assessment of HKA and MAD before the operation provided key data support for the design of the osteotomy plan, ensuring that the lower extremity force line returned to the physiological range (HKA 180°±2°, MAD ± 10 mm). Furthermore, biplanar osteotomy maintains the blood supply and stability at the osteotomy end by preserving the integrity of the lateral cortex (with a thickness of 5–10 mm), which is in line with the principle of biomechanical optimization. Schematic diagram illustrating the biplanar distal femoral closed-wedge osteotomy technique used. The transverse osteotomy extends approximately 3-quarters across the distal femur. The coronal osteotomy is oriented at an angle of 100° relative to the transverse cut. The planned wedge depth (5.6 mm) and correction angle (5.6°) correspond to the preoperative calculations based on Hernigou trigonometric correction chart (2010). Illustration depicts measurement principles; intraoperative planning and postoperative assessment utilized picture archiving and communication system system electronic angle measurement tools (Fig. 11).

The interaction between osteotomy correction and ACL graft biomechanics warrants detailed analysis. Of particular relevance is the effect of sagittal plane alignment changes—specifically femoral condylar torsion (posterior slope angle)–on ACL graft stress. Computational biomechanical studies by Phillips et al (2022) demonstrated that increased femoral posterior slope (>12°) significantly elevates ACL graft tensile forces during weight-bearing flexion. Their multibody knee dynamics model predicted a 15% to 28% increase in peak ACL graft stress per 5° increase in posterior slope, primarily due to altered tibiofemoral shear forces during mid-flexion activities. Complementary cadaveric studies further validate this relationship: Rahnemai-Azar et al^[18]^ reported a 2.1-mm increase in anterior tibial translation per 5° increase in posterior slope under 134-N anterior load, directly amplifying ACL graft strain.

In the present case, the biplanar closed-wedge design intentionally preserved sagittal alignment. Preoperative lateral radiographs confirmed physiologic posterior slope (3.2°). Hernigou method ensured isolated coronal correction (ΔHKA = 4.58°) without altering the sagittal plane, as verified by postoperative lateral fluoroscopy (Fig. 3B; slope = 3.5°±1°). This technical precision prevented pathologic graft loading, as excessive posterior slope reduction (<0°) may increase graft strain during hyperextension, while slope elevation (>10°) exacerbates mid-flexion shear forces.^[19]^

The biomechanical coupling between MPFL reconstruction and ACL graft loading constitutes a critical factor influencing clinical outcomes in combined procedures. Finite element modeling by Lorbach et al^[20]^ demonstrated that the anatomical reconstruction of MPFL could significantly reduce the contact pressure of the patellofemoral joint. At a 30° knee flexion, both the femoris thin muscle graft group and the latissimus fascia graft group showed that the contact pressure distribution was close to that of the normal knee joint. Among them, the latissimus fascia graft reduced the pressure by 23% to 37% when the knee flexion was 45°–90°. Through the above-mentioned method of dynamically monitoring the distance between the 2 marking needles under intraoperative fluoroscopy guidance (simulating the length of the graft), it was measured that the length variation of the MPFL graft within the entire range of knee flexion movement from 0° to 90° was 1.4 ± 0.3 millimeters. In our case, intraoperative isometric assessment of the MPFL graft via fluoroscopic tracking of radiopaque markers confirmed minimal length change (1.4 ± 0.3 mm) during 0° to 90° flexion, with optimal tensioning achieved at 30° flexion. This corresponds to the biomechanical sweet spot identified by Victor et al^[6]^ where MPFL tensile forces peak at 30° flexion, necessitating precise tensioning to balance patellar stability and ACL graft protection. The tension equilibrium between ACL and MPFL grafts was further optimized through anatomical referencing. The MPFL femoral tunnel was positioned 10 to 15 mm distal to the ACL femoral footprint, aligning with the isometric zones reported by Felli et al^[21]^ where 30° flexion provides optimal length-tension relationship for both structures. However, this biomechanical optimization requires careful consideration of osteotomy-induced alignment changes. Phillips et al (2022) demonstrated that increased femoral posterior slope (>12°) elevates ACL graft stress by 15% to 28% per 5° increment. Our biplanar osteotomy preserved native posterior slope (3.2° preop vs 3.5° postop), preventing secondary graft loading. This aligns with the “sagittal plane neutrality” principle emphasized by Rahnemai-Azar et al^[18]^ to mitigate shear forces on ACL grafts post-osteotomy. Intraoperative isometric assessment of the MPFL graft (performed via fluoroscopic tracking of radiopaque markers) confirmed minimal length change (1.4 ± 0.3 mm) during 0° to 90° flexion, with optimal tensioning at 30°.^[22]^

Valgus deformity increases the *Q*-angle and imposes a laterally directed force vector on the patella, contributing to lateral tracking, instability, and elevated lateral facet contact pressures. Correction of the valgus alignment via DFO directly reduces this lateralizing force vector and improves the overall biomechanical environment of the patellofemoral joint. As demonstrated by Lorbach et al^[20]^ using finite element analysis, anatomical MPFL reconstruction alone significantly reduces patellofemoral contact pressures and restores near-normal pressure distribution. It is mechanistically sound to infer that combining MPFL reconstruction with corrective osteotomy to address the underlying osseous deformity would provide a more comprehensive and durable normalization of patellofemoral contact mechanics, potentially reducing the long-term risk of patellofemoral osteoarthritis associated with chronic instability and malalignment. Stephen et al,^[22]^ utilizing a cadaveric model simulating patellar instability, further showed that MPFL reconstruction effectively reduced excessive lateral patellar shift and tilt, key determinants of abnormal contact pressures. Therefore, the combined approach employed in this case – rectifying the valgus malalignment and reconstructing the MPFL – targets both the primary osseous driver and the deficient soft-tissue restraint of patellar instability, theoretically offering superior long-term protection to the patellofemoral articular cartilage by normalizing contact pressures compared to addressing either pathology in isolation. Long-term cohort studies or registry data specifically focusing on combined DFO and MPFL reconstruction would be valuable to confirm these biomechanically predicted benefits on patellofemoral joint preservation.

Osteotomy procedures may result in the emergence of secondary deformities, characterized by joint line tilt in various directions, which subsequently influences the shear force experienced by the knee joint. Prior to surgical intervention, standard X-rays should be employed to assess the line of force in the lower limb, taking into account the proximal angle of the medial tibia, the distal angle of the lateral femur, and the inclination of the joint line (Table 3). The primary objective of osteotomy is to establish the desired line of force while ensuring that the joint line remains free of tilt. In this context, the inverse Miniaci method is utilized to measure the angle, and the osteotomy distance should be adjusted in accordance with Hernigou table.

**Table 3:** Required depth of osteotomy measured at different osteotomy diameters and angles of correction.

Trigonometric chart																	
	Correction angle																
Mediolateral diameter (D) of the osteotomy (mm)		4°	5°	6°	7°	8°	9°	10°	11°	12°	13°	14°	15°	16°	17°	18°	19°
	50	3	4	5	6	7	8	9	10	10	11	12	13	14	15	16	16
	55	4	5	6	7	8	9	10	10	11	12	13	14	15	16	17	18
	60	4	5	6	7	8	9	10	11	12	14	15	16	17	18	19	20
	65	5	6	7	8	9	10	11	12	14	15	16	17	18	19	20	21
	70	5	6	7	8	10	11	12	13	15	16	17	18	20	21	22	23
	75	5	6	8	9	10	12	13	14	16	17	18	20	21	22	24	25
	80	6	7	8	10	11	13	14	15	17	18	19	21	22	24	25	26

## Acknowledgments

I sincerely thank the Library of Binzhou Medical College and the Affiliated Hospital of Binzhou Medical University for providing the electronic data resource platform, and also thank all the teachers and students who have given valuable advice and guidance.

## Author contributions

**Conceptualization:** Hui Shi.

**Data curation:** Heng Cao, Jian Li.

**Formal analysis:** Heng Cao.

**Investigation:** Yao-Min Wang, Heng Cao, Xinlong Chen.

**Methodology:** Hui Shi.

**Resources:** Heng Cao.

**Writing – original draft:** Yao-Min Wang, Heng Cao.

**Writing – review & editing:** Yao-Min Wang, Qiang Ren, Hui Shi.
